# A randomized, double-blind, placebo-controlled, phase IIa, clinical study on investigating the efficacy and safety of SPH3127 tablet in patients with essential hypertension

**DOI:** 10.1038/s41440-024-01657-z

**Published:** 2024-04-17

**Authors:** Fang Wang, Ling Liu, Hongyun Ruan, Xiaoping Chen, Yue Zhang, Zaixin Yu, Yuhui Li, Yang Guan, Jiguang Wang, Kai Huang, Shunjiang Yu, Yuanyuan Cao, Cungang Ding, Lin Chang, Yaohua Huang, Xiangjuan Chen, Qiang Lv, Changsheng Ma

**Affiliations:** 1 Department of Cardiology, Beijing Hospital, No. 1 Dahua Road, Dongcheng District, Beijing, China; 2 Department of Cardiovascular Medicine, The Second Xiangya Hospital of Central South University, No. 139, Renmin Middle Road, Changsha, Hunan Province China; 3 Department of Cardiology, Xuzhou Central Hospital, No. 199 Jiefang South Road, Quanshan District, Xuzhou, Jiangsu Province, China; 4 Department of Cardiology, West China Hospital of Sichuan University, No. 37 Guoxue Lane, Wuhou District, Chengdu, Sichuan Province China; 5 Department of Cardiology, The affiliated Hospital of inner Mongolia Medical University, No.1, Tongtong North Street, Huimin District, Hohhot, Inner Mongolia Autonomous Region China; 6 Department of Cardiology, Xiangya Hospital Central South University, No. 87, Xiangya Road, Kaifu District, Changsha, Hunan Province China; 7 Department of Cardiology 1, Guangdong Second Provincial Central Hospital, No. 466, Xingang Middle Road, Haizhu District, Guangzhou, Guangdong Province China; 8 Department of Cardiology 2, Beijing Anzhen Hospital, Capital Medical University, No. 2 Anzhen Road, Chaoyang District, Beijing, China; 9 Department of Hypertension, Ruijin Hospital Affiliated to The Shanghai Jiao Tong University Medical School, No. 197, Ruijin 2nd Road, Huangpu District, Shanghai, China; 10 Department of Cardiology, Union Hospital Tongji Medial College Huazhong University of Science and Technology, No. 1277 Jiefang Avenue, Wuhan City, Hubei Province China; 11 Department of New Drug Registration and Clinical R&D, Shanghai Pharmaceuticals Holding Co. Ltd, No. 200 Taicang Road, Huangpu District, Shanghai, China

**Keywords:** Cardiovascular, Efficacy, Essential hypertension, Safety, SPH3127

## Abstract

Around 70% of patients diagnosed with hypertension exhibit increased levels of renin. SPH3127, an inventive renin inhibitor, has shown favorable tolerability and sustained pharmacodynamic inhibitory impact on plasma renin activity (PRA) during previous phase I trials. This phase II study was conducted to investigate the efficacy and safety of SPH3127 in patients with essential hypertension. This study was conducted in patients with mild to moderate essential hypertension, utilizing a randomized, double-blind, placebo-controlled design. The patients were administered either tablet of SPH3127 at doses of 50 mg, 100 mg, or 200 mg, or a placebo. A total of 122 patients were included in the study, with 121 patients included in the full analysis set. Among these patients, there were 30 individuals in each subgroup receiving different dosage regimens of SPH3127, and 31 patients in the placebo group. The reductions in mean sitting diastolic blood pressure (msDBP) after 8 weeks compared to baseline were 5.7 ± 9.5, 8.6 ± 8.8, and 3.8 ± 10.6 mmHg in the SPH3127 50-, 100-, and 200 mg groups, respectively. In the placebo group, the reduction was 3.1 ± 8.4 mmHg. The corresponding reductions in mean sitting systolic blood pressure (msSBP) were 11.8 ± 13.0, 13.8 ± 11.2, 11.1 ± 13.1, and 7.7 ± 9.7 mmHg in each respective group. SPH3127 is a promising drug for the treatment of patients with essential hypertension. The recommended dosage is 100 mg daily.

Clinical trial registration: This study was registered in ClinicalTrials.gov (NCT03756103).

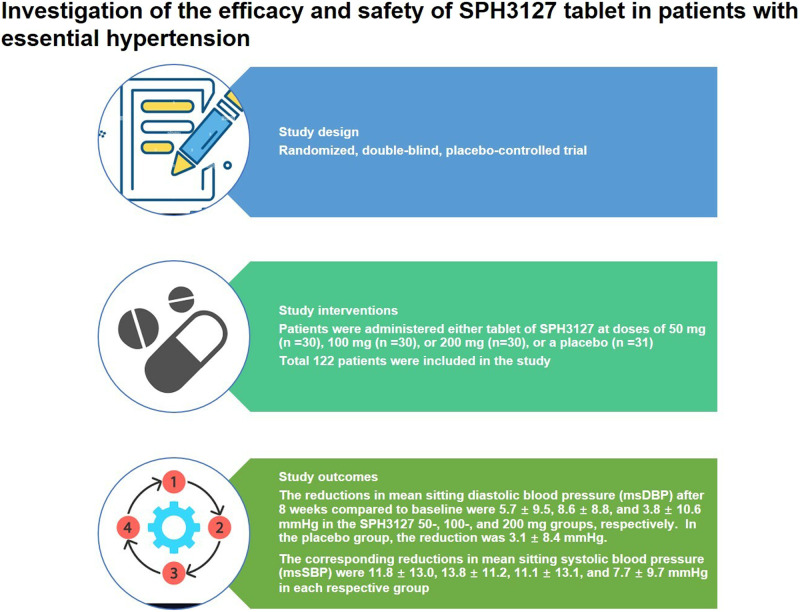

## Introduction

The etiology and progression mechanisms of essential hypertension are extremely complex. In addition to various factors such as vascular function, salt intake, activation of the neuroendocrine system, and genetic alteration, inflammation and oxidative stress may also play a role, with multiple factors intertwining and influencing each other [1]. It is known that the renin-angiotensin-aldosterone system (RAAS) primarily regulates blood pressure (BP) by affecting arterial constriction and intracellular sodium and water retention. During this process, the RAAS regulates the pathogenesis of hypertension and organ damage throughout the blood, heart, blood vessels, kidneys, brain, and adipose tissues [2]. It is well known that excessive activation of the RAAS leads to vessel tension. Consequently, RAAS inhibitors have become the cornerstone of antihypertensive medications. This first-line choice for antihypertensive treatment is complicated if considering the potential target organ damage such as heart failure and possible renal impairment.

Approximately 70% of patients with hypertension have elevated level of renin [3]. Renin is mainly secreted by juxtaglomerular cells in the kidney and serves as a specific rate-limiting enzyme. Once released into the bloodstream, it converts angiotensinogen (AGT) into angiotensin (Ang) I and activates the entire RAAS. As renin is the only known enzyme capable of cleaving AGT, which is the sole substrate in the RAAS that generates all downstream Ang peptides, renin inhibitors can block the cascade reaction of the RAAS and lower BP by reducing the generation of Ang I from the upstream [4]. Therefore, the agent targeting direct renin inhibition is a potential superior choice for RAAS blockade downstream.

Aliskiren is a highly potent and selective renin inhibitor with an elimination half-life of 40 h. It can efficaciously lower BP within 24 h when administered once daily [3, 5], either as monotherapy or in combination with other antihypertensive drugs [3, 6, 7]. Although it can be administered in combination with angiotensin-converting enzyme inhibitors (ACEIs) or angiotensin receptor blockers (ARBs), it possibly increases the risk of hyperkalemia [8].

SPH3127 has been discovered as a novel, oral, non-peptide, renin inhibitor with a similar mechanism of Aliskiren [9]. Even though SPH3127 has a relatively lower molecular weight (MW) in comparison to Aliskiren, it efficiently forms hydrophobic interactions and six hydrogen bonds with renin as demonstrated by X-ray crystallography [9]. Furthermore, SPH3127 exhibited higher bioactivity with more potent antihypertensive effect in preclinical models than Aliskiren due to its unique chemical structure containing methyl substituents to improve renin inhibitory activity and the replacement of the indole skeleton by 2,7-diazaindole to improve the pharmacokinetic profile [9]. In earlier phase I studies, SPH3127 has demonstrated good tolerability and a more potent and long-lasting inhibitory effect on plasma renin activity (PRA) [10].

This phase IIa, multicenter, randomized, double-blind, placebo-controlled, dose exploration study evaluated the efficacy and safety of different dosage regimens (50, 100, and 200 mg) of SPH3127 tablet for the treatment of patients with mild to moderate essential hypertension.

## Methods

### Patients

Patients aged 18–65 years with mild to moderate essential hypertension who also met the following criteria were enrolled in this study: (1) mean sitting diastolic blood pressure (msDBP) ≥ 90 and ≤109 mmHg before randomization, (2) mean sitting systolic blood pressure (msSBP) ≥ 140 and ≤179 mmHg before randomization, and (3) laboratory tests: GFR ≥ 60 mL/min, AST or ALT < 2 times of upper limit of the normal, hemoglobin ≥90 g/L, and so on. Patients with secondary and/or malignant hypertension, structural or functional heart disease within 6 months, cerebrovascular disease within 6 months, uncontrolled diabetes [fasting blood glucose >7.8 mmol/L or glycosylated hemoglobin (HbA1c) > 7.5%], malignant retinopathy, history of mental disorder, history of malignant tumor, alcohol or drug abuse, and those receiving concomitant drugs with potential effect on BP were excluded from the study. Pregnant, lactating women or subjects who is planning to pregnant within 6 months after the trial were also excluded. All patients should sign the consent form before screening.

### Design

This study was a randomized, double-blinded, placebo-controlled study. Ten centers from China participated in this study. All random schemes were digitalized from the independent statistical professionals with SAS software, which were sealed and then securely held by the investigator-leader unit and sponsor. The labeling of drugs and the procedure of urgent unbinding in the Interactive Web Response System (IWRS) were prepared by the staffs who were unrelated to the study of any commercial benefits. The appearance and packaging except drug number between the tested drug and the placebo were identical, and investigators and patients were never able to identify the specific drugs used. This study was submitted to ethical institutions and notified with formal approval in compliance with the Declaration of Helsinki and the Clinical Practice Guidelines during human medical study. This study was registered with ClinicalTrials.gov (NCT03756103).

### Study procedure

**Fig. 1:**
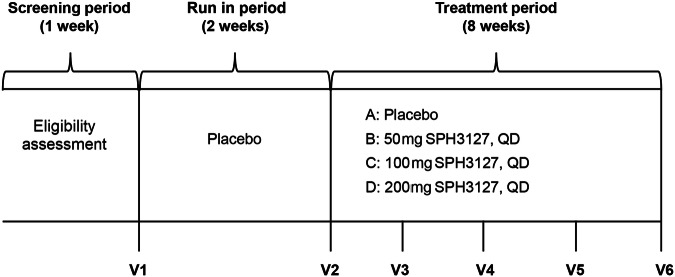
Flowchart of the study. QD once a day

This study consists of 3 treatment periods: 1 week of screening period, 2 weeks of run-in period (administering with 4 tablets of placebo), and 8 weeks of double-blinded, placebo-controlled, treatment period. Eligible patients were randomly allocated (1:1:1:1) to a once-daily regimen as presented in Fig. 1.

Sitting blood pressure was measured thrice by one physician in examination room for each patient between 8 am to 12 am before treatment on every visit and the mean value was calculated. During this study, half of the patients were monitored using a 24-h ambulatory BP monitor twice (24 h before the 2^nd^ an 6^th^ visit) and using additional PRA tests thrice (on the 2^nd^, 4^th^ and 6^th^ visit). Briefly, plasma samples were separately incubated with renin substrate angiotensinogen at 37 °C and 0 °C (pH = 6.0) for 2 h to generate angiotensin enzyme I (Ang-I). A validated enzyme-linked immunosorbent assay (ELISA) was used to determine the concentration of Ang-I and PRA was calculated using the following formula: = ([Ang-I (37 °C)] − [Ang-I (0 °C)])/(Time (2 h)) × 1.11. AEs, physical examinations and laboratory tests were recorded as safety assessment.

### End points

The primary efficacy end point of this study was to assess the changes in msSBP and msDBP from baseline to 8 weeks. The secondary efficacy end points included (1) changes in msSBP and msDBP from baseline to 2, 4, and 6 weeks of treatment; (2) changes in the 24-h ambulatory BP (DBP and SBP throughout the day) from baseline to 8 weeks of treatment; (3) marked response rate (defined as msDBP decreased to ≥10 mmHg and within the normal range or decreased to ≥20 mmHg but not within the normal range or msSBP decreased to ≥30 mmHg), response rate (defined as msDBP decreased to <10 mmHg and within the normal range or decreased to 10–19 mmHg but not within the normal range or the msSBP decreased to ≥20 mmHg), the total response rate (the sum of marked response rate and response rate after 4 and 8 weeks of treatment); (4) the control rate after 4 and 8 weeks of treatment, defined as the proportion of patients whose msDBP was <90 mmHg and msSBP was <140 mmHg; and (5) changes in PRA from baseline to 4 and 8 weeks of treatment.

Safety was reported throughout the study, and all AEs, including the serious adverse events (SAEs), were documented. The AEs were categorized according to CTCAE version 4.03. The important AE was defined as an AE with a severity of grade 3 or more and/or that led to discontinuation of SPH3127 (the re-dosing after discontinuation or permanent discontinuation).

### Statistical analyses

As this was a phase IIa exploratory study, the sample size was not statistically calculated. The 3 different dosage regimens of SPH3127 tablet and the placebo were presumably allocated with 30 patients in each group, with an approximate dropout rate of <20%. The full analysis set (FAS) included all the patients who were randomized and received at least 1 dose of the investigational drug. The per-protocol set (PPS) was a subset of FAS that included patients with good compliance and no major protocol deviation. An efficacy analysis was performed both on FAS and PPS; the safety set (SS) included patients who received at least 1 dose of the investigational drug. The last observation carry forward (LOCF) method was used to handle missing data.

Statistical analyses were carried out using SAS 9.4. A two-sided test with a 95% confidence level (CI) was adopted at a significance level of α = 0.05. Continuous variables were displayed using case number, missing number, mean, standard deviation (SD). Categorical variables were expressed as number and percentage. Analysis of variance (ANOVA) and paired *t* test was used for inter-group and intra-group comparison of difference values (D-value) and change rate before and after treatment, respectively. With respect to the primary efficacious end points data, the center effect was integrated during the ANOVA. The chi-square test or Fisher’s exact probability were used to analyze the incidence rates of safety parameters.

## Results

### Demographics and baseline characteristics

A total of 122 patients were randomized into 4 groups (Supplementary Fig. 1), of which 121 patients were included in the FAS, including 30 patients in each subgroup of the 3 different dosage regimens of SPH3127 tablet and 31 patients in the placebo group. Demographics and baseline characteristics were well balanced in the 4 groups (Table 1). A total of 108 patients were included in the PPS, including 26 patients in the SPH3127 50-mg group, 24 patients in the 100-mg group, 27 patients in the 200-mg group, and 31 patients in the placebo group (Supplementary Fig. 1).

**Table 1:** Demographic and baseline characteristics

	Placebo (*n* = 31)	SPH3127 50 mg (*n* = 30)	SPH3127 100 mg (*n* = 30)	SPH3127 200 mg (*n* = 30)
Gender, *n* (%)				
Male	20 (64.5)	16 (53.3)	20 (66.7)	20 (66.7)
Female	11 (35.5)	14 (46.7)	10 (33.3)	10 (33.3)
Age, mean (SD), y	49.3 (9.5)	49.9 (8.2)	48.6 (11.2)	48.0 (9.5)
BMI, mean (SD), kg/m^2^	26.4 (3.2)	25.8 (3.4)	26.6 (3.5)	26.7 (3.8)
msDBP, mean (SD), mmHg	91.5 (8.8)	89.5 (9.4)	91.6 (9.2)	90.2 (9.6)
msSBP, mean (SD), mmHg	146.7 (7.0)	148.0 (7.2)	147.6 (9.4)	147.4 (5.4)

Continuous variables were denoted as mean (SD); categorical variables were denoted as *n* (%)

*BMI* body mass index, *msDBP* mean sitting diastolic blood pressure, *msSBP* mean sitting systolic blood pressure, *SD* standard deviation

All patients were reconfirmed to have mild or moderate essential hypertension before randomization. The previous antihypertensive medications were roughly proportional in the 4 groups: 10 (31.3%) patients in the placebo group, 12 (40.0%) patients in the 50-mg group, 13 (43.3%) patients in the 100-mg group, and 17 (56.7%) patients in the 200-mg group.

### Efficacy

#### Primary endpoints

##### msDBP

In the FAS, after 8 weeks of treatment, the msDBP was unanimously decreased from the baseline by 5.7 ± 9.5, 8.6 ± 8.8, and 3.8 ± 10.6 mmHg in the SPH3127 50-, 100-, and 200-mg groups, respectively, and 3.1 ± 8.4 mmHg in the placebo group.

In the PPS, after 8 weeks of treatment, the msDBP decreased by 5.6 ± 10.2, 10.0 ± 9.2, and 4.0 ± 10.2 mmHg in the SPH3127 50-, 100-, and 200-mg groups, respectively, and 3.1 ± 8.4 mmHg in the placebo group.

The sensitive analysis of the PPS also suggested that the 100-mg dosage was associated with the greatest reductions in msDBP; on the other hand, placebo was associated with the smallest reduction (Table 2).

**Table 2:** Changes in msDBP from baseline to 8 weeks of treatment

	FAS				PPS			
	Placebo (*n* = 31)	SPH3127 50 mg (*n* = 30)	SPH3127 100 mg (*n* = 30)	SPH3127 200 mg (*n* = 30)	Placebo (*n* = 31)	SPH3127 50 mg (*n* = 26)	SPH3127 100 mg (*n* = 24)	SPH3127 200 mg (*n* = 27)
Pre-dosing								
Mean (SD)	91.5 (8.8)	89.5 (9.4)	91.6 (9.2)	90.2 (9.6)	91.5 (8.8)	88.9 (8.8)	91.9 (8.3)	89.7 (10.0)
8 weeks								
Mean (SD)	88.3 (10.0)	83.9 (12.3)	83.0 (8.7)	86.4 (10.3)	88.3 (10.0)	83.3 (12.5)	81.9 (8.4)	85.7 (10.1)
msDBP changes								
Mean (SD)	−3.1 (8.4)	−5.7 (9.5)	−8.6 (8.8)	−3.8 (10.6)	−3.1 (8.4)	−5.6 (10.2)	−10.0 (9.2)	−4.0 (10.2)

Bold indicates that the difference between pre- and post-administration intra-groups is of statistical significance

*msDBP* mean sitting diastolic blood pressure, *SD* standard deviation

##### msSBP

In the FAS, after 8 weeks of treatment, the msSBP decreased from baseline by 11.8 ± 13.0, 13.8 ± 11.2, and 11.1 ± 13.1 mmHg in the SPH3127 50-, 100-, and 200-mg groups, respectively, and 7.7 ± 9.7 mmHg in the placebo group.

In the PPS, after 8 weeks of treatment, the msSBP decreased from baseline by 12.2 ± 13.8, 16.2 ± 10.8, and 12.3 ± 11.9 mmHg in the SPH3127 50-, 100-, and 200-mg groups, respectively, and 7.7 ± 9.7 mmHg in the placebo group.

The reductions in msSBP were greater in the SPH3127 100-mg group compared with other dosage groups and placebo group, whereas similar reductions were reported with 50- and 200-mg dosages (Table 3).

**Table 3:** Changes in msSBP from baseline to 8 weeks of treatment

	FAS				PPS			
	Placebo (*n* = 31)	SPH3127 50 mg (*n* = 30)	SPH3127 100 mg (*n* = 30)	SPH3127 200 mg (*n* = 30)	Placebo (*n* = 31)	SPH3127 50 mg (*n* = 26)	SPH3127 100 mg (*n* = 24)	SPH3127 200 mg (*n* = 27)
Pre-dosing								
Mean (SD)	146.7 (7.0)	148.0 (7.2)	147.6 (9.4)	147.4 (5.4)	146.7 (7.0)	147.1 (7.0)	146.8 (8.8)	147.2 (5.4)
8 weeks								
Mean (SD)	139.1 (11.1)	136.2 (14.5)	133.9 (12.8)	136.3 (11.5)	139.1 (11.1)	134.9 (15.0)	130.7 (12.0)	135.0 (9.8)
msSBP changes								
Mean (SD)	−7.7 (9.7)	−11.8 (13.0)	−13.8 (11.2)	−11.1 (13.1)	−7.7 (9.7)	−12.2 (13.8)	−16.2 (10.8)	−12.3 (11.9)

Bold indicates that the difference between pre- and post-administration intra-groups is of statistical significance

*msSBP* mean sitting systolic blood pressure, *SD* standard deviation

From the primary analysis, SPH3127 tablet is efficacious to decrease the msDBP and msSBP after 8 weeks of treatment with each dosage regimen compared with placebo. Moreover, the reductions in BP were greater in the SPH3127 100-mg group compared with other dosage groups and placebo group.

#### Secondary end points

##### Changes in msDBP and msSBP from baseline to 2, 4, and 6 weeks of treatment

**Fig. 2:**
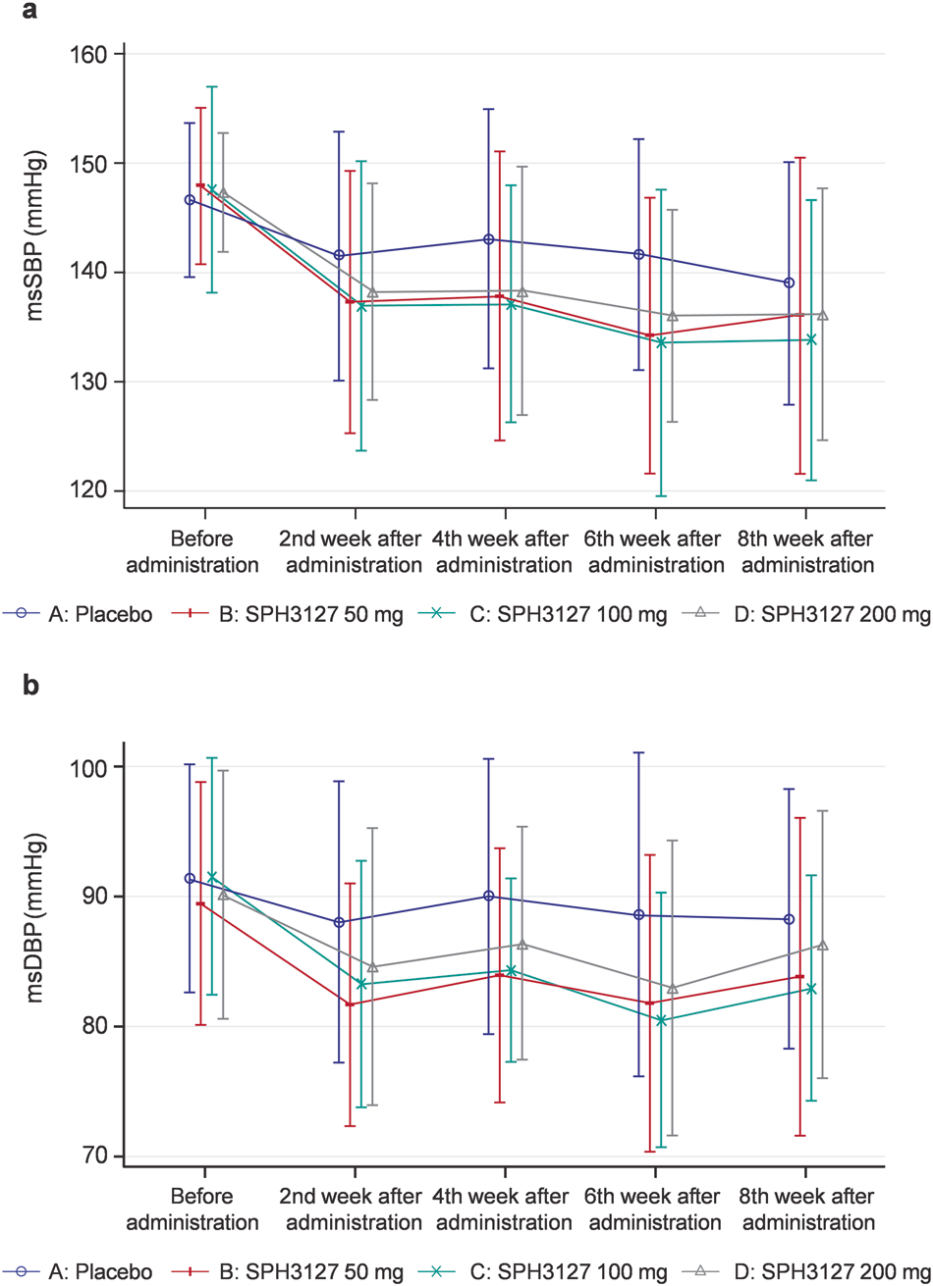
Changes in msDBP and msSBP during the consecutive follow-ups. msDBP mean sitting diastolic blood pressure, msSBP mean sitting systolic blood pressure

The changes in the msDBP and msSBP from baseline to 2, 4, and 6 weeks of treatment showed a similar trend of decrease in the SPH3127 dosage groups, and the decrease was greater compared with that in the placebo group (Supplementary Fig. 1 and Fig. 2). In particular, in the SPH3127 100-mg group, the biweekly measurements clearly showed that the mean reductions in msBP were evident by week 2, with further reductions at week 6, exhibiting the most obvious downward trend of both msSBP and msDBP over time (Fig. 2).

##### Changes in 24-h ambulatory BP after 8 weeks of treatment

After 8 weeks of treatment, the changes in the 24-hour ambulatory msDBP were 1.15, 4.19, 3.86, and 4.70 mmHg and the changes in msSBP were 0.06, 5.15, 7.32, and 15.38 mmHg in the placebo and SPH3127 50-, 100-, as well as 200-mg groups, respectively. Generally, the decrease in 24-hour ambulatory BP was most evident in the 100-mg group, although all the 3 dosages reduced either msDBP or msSBP in varying degrees (Supplementary Fig. 2).

##### Response rate and control rate after 4 and 8 weeks of treatment

Both total response rate and control rate in the 3 SPH3127 dosage groups were all higher than those in the placebo group after 4 and 8 weeks of treatment, whereas the most remarkable efficacy was reported in the 100-mg group (Supplementary Table 1).

##### Changes in PRA from baseline after 4 and 8 weeks of treatment

The most significant decrease in PRA was reported in the SPH3127 100-mg group after 8 weeks of treatment, and both the 100- and 200-mg groups were reported with a decrease in PRA from baseline after 4 and 8 weeks of treatment, whereas no significant change was observed in the placebo group (Supplementary Fig. 3).

### Safety analysis

In the SS of 122 patients, AEs were reported by 17 (56.67%), 22 (73.33%), 23 (76.67%), and 26 (81.25%) patients in the SPH3127 50-, 100-, and 200-mg tablet and placebo groups, respectively, and the total number of AEs reported was 52, 73, 61, and 85 in each group, respectively (Table 4).

**Table 4:** Safety analysis of 3 dosage regimens of SPH3127 versus placebo

	Placebo (*n* = 32)	SPH3127 50 mg (*n* = 30)	SPH3127 100 mg (*n* = 30)	SPH3127 200 mg (*n* = 30)
AEs	26 (81.25%)	17 (56.67%)	22 (73.33%)	23 (76.67%)
Important AEs	1 (3.13%)	2 (6.67%)	1 (3.33%)	1 (3.33%)
CTCAE ≥ 3	1 (3.13%)	2 (6.67%)	1 (3.33%)	1 (3.33%)
AEs leading to discontinuation	0 (0.00%)	2 (6.67%)	0 (0.00%)	1 (3.33%)
SAE	0 (0.00%)	0 (0.00%)	1 (3.33%)	0 (0.00%)
AEs (or probably) related to the treatment	5 (15.63%)	3 (10.00%)	6 (20.00%)	7 (23.33%)
Elevated BP	0 (0.00%)	1 (3.33%)	0 (0.00%)	2 (6.67%)
Elevated ALT	0 (0.00%)	0 (0.00%)	1 (3.33%)	1 (3.33%)
Urine protein	1 (3.13%)	0 (0.00%)	0 (0.00%)	0 (0.00%)
Elevated eosinophils	0 (0.00%)	0 (0.00%)	1 (3.33%)	0 (0.00%)
Elevated AST	0 (0.00%)	0 (0.00%)	1 (3.33%)	0 (0.00%)
Elevated triglyceride	0 (0.00%)	0 (0.00%)	1 (3.33%)	0 (0.00%)
Decreased serum phosphate	1 (3.13%)	0 (0.00%)	0 (0.00%)	0 (0.00%)
Elevated uric acid	0 (0.00%)	0 (0.00%)	1 (3.33%)	0 (0.00%)
Dizziness	2 (6.25%)	0 (0.00%)	1 (3.33%)	1 (3.33%)
Headache	1 (3.13%)	0 (0.00%)	0 (0.00%)	1 (3.33%)
Bradycardia	0 (0.00%)	0 (0.00%)	1 (3.33%)	0 (0.00%)

*AE* adverse event, *BP* blood pressure, *SAE* serious adverse event, *AST* aspartate aminotransferase, *ALT* alanine transaminase

Most of AEs were grades 1 to 2 in severity, and AEs of grade 3 or more were reported by 2 (6.67%), 1 (3.33%), 1 (3.33%) and 1 (3.13%) patients in SPH3127 50-, 100-, and 200-mg and the placebo groups, respectively. Two AEs of liver injury and hypertension in the 50-mg group led to dosing discontinuation. One AE of aggravated hypertension was observed in the 200-mg group leading to the discontinuation. However, with respect to these important AEs, no significant differences were observed in the 4 groups. In addition, all the important AEs were manageable, being curative after discontinuation of interventions, and no sequelae was reported during the follow-up.

Probability of AEs associated with SPH3127 were reported in 3 (10%) patients with 3 AEs in the 50-mg group, 6 (20%) patients with 9 AEs in the 100-mg group, 7 (23.3%) patients with 9 AEs in the 200-mg group and 5 (15.63%) patients with 6 AEs in the placebo group. Most AEs were abnormal laboratory values and recovered without any intervention (Table 4).

## Discussion

This exploratory study evaluated the efficacy and safety of different dosage regimens (50, 100, and 200 mg) of SPH3127 tablet for the treatment of patients with mild to moderate essential hypertension. The result showed that both msSBP and msDBP decreased significantly from baseline to 2, 4, 6, and 8 weeks after treatment with the 3 dosage regimens of SPH3127 tablet compared with placebo. There were more remarkable decreases in both msSBP and msDBP of the 100-mg group after 8 weeks of treatment comparing with the other dosage regimens of SPH3127. Analyses such as 24-hour ambulatory BP monitoring, control rates for target BP, total response rates and changes in PRA all suggested a favorable clinical outcome in the SPH3127 100-mg group.

With respect to the placebo-controlled study with a duration of not less than 8 weeks for the same class drug of Aliskiren, the changes in the msSBP and msDBP from baseline were 8.72–13 and 7.5–10.3 mmHg, respectively, with a dosage regimen of 150 mg daily [11]. In this SPH3127 phase IIa study, the SPH3127 100-mg tablet group showed an average reduction of 13.77 and 8.64 mmHg in the msSBP and msDBP, respectively, which was similar to that observed in Aliskiren studies.

On the other hand, for Aliskiren 150-mg group, the control rates were 48.11% [12] and 35.9% [13] from the study conducted in Asia and the United States, respectively, and the corresponding DBP response rates were 59.75% [12] and 59.3% [13]. The control rate (63.33%) and total response rate (80.00%) for the SPH3127 100-mg group were similar to those reported in Aliskiren studies.

As RAAS inhibitors, both ACEIs and ARBs could lead to an increase in PRA, whereas Aliskiren leads to a decrease in PRA [14]. Earlier phase I study demonstrated that PRA could be inhibited in 90% of healthy volunteers administered with a single dose of SPH3127 ≥ 100-mg tablet and the effect could last for >24 h. In this phase IIa study, the decrease in PRA was also observed in the 100- and 200-mg groups after 4 and 8 weeks of treatment.

In a meta-analysis, 6 short-term studies with a duration of up to 8 weeks were pooled, and the frequency of AEs associated with Aliskiren 150 mg was reported as 33.6%, which was similar to that observed in the placebo group (36.8%) [15]. The frequency of AEs of special interest including vascular edema (0.2%), hyperkalemia (0.0%), and diarrhea (1.4%) was also similar to that observed in the placebo group [15]. In a randomized, double-blind, placebo-controlled, 8-week study, the most common adverse effects associated with Aliskiren included headache (2.4%), dizziness (2.9%), and diarrhea (2.5%) [14, 16]. No AEs of special interest associated with SPH3127 were observed in the present study, and the frequency of treatment-related AEs (17.78%; 16/90) was similar to that observed in the placebo group (15.63%). Except for one case of hypertension exacerbation categorized as CTCAE grade 3, the severity of other AEs, such as abnormal liver function and dizziness, was CTCAE grade 1 or 2. Most AEs were resolved spontaneously without any sequelae. Overall, SPH3127 is safe for the treatment of patients with mild to moderate hypertension.

The limitations of this phase IIa study are the small sample size, basically presumed on empirical considerations, and a shortage of extensive follow-up visits during treatment and observational periods. Nevertheless, these are inherent characteristics of phase IIa study, which was actually intended to preliminarily investigate antihypertensive effect as well as safety and to choose an appropriate dose for future clinical studies.

This study showed that SPH3127 tablet was effective in treating patients with mild to moderate hypertension in a dosage regimen of 50, 100, or 200 mg daily compared with placebo. The optimal dosage from efficacy’s perspective is 100 mg. In addition, SPH3127 is safe and well tolerated in all 3 dosage regimens; therefore, SPH3127 100 mg daily is the dosage recommended for future clinical studies.

### Supplementary information

Supplementary Information
