# Supplementary material for: A randomized, double-blind, placebo-controlled, phase IIa, clinical study on investigating the efficacy and safety of SPH3127 tablet in patients with essential hypertension
Source: Hypertens Res. 2024 Apr 17;47(7):1925–33. doi: 10.1038/s41440-024-01657-z (PMC11224011; doi:10.1038/s41440-024-01657-z)
Supplement: Supplementary file 1 — Supplementary Information [file 41440_2024_1657_MOESM1_ESM.docx]

**Supplementary Fig. 1** CONSORT patient flow diagram Major protocol deviation leading to exclusion from Per protocol set

**
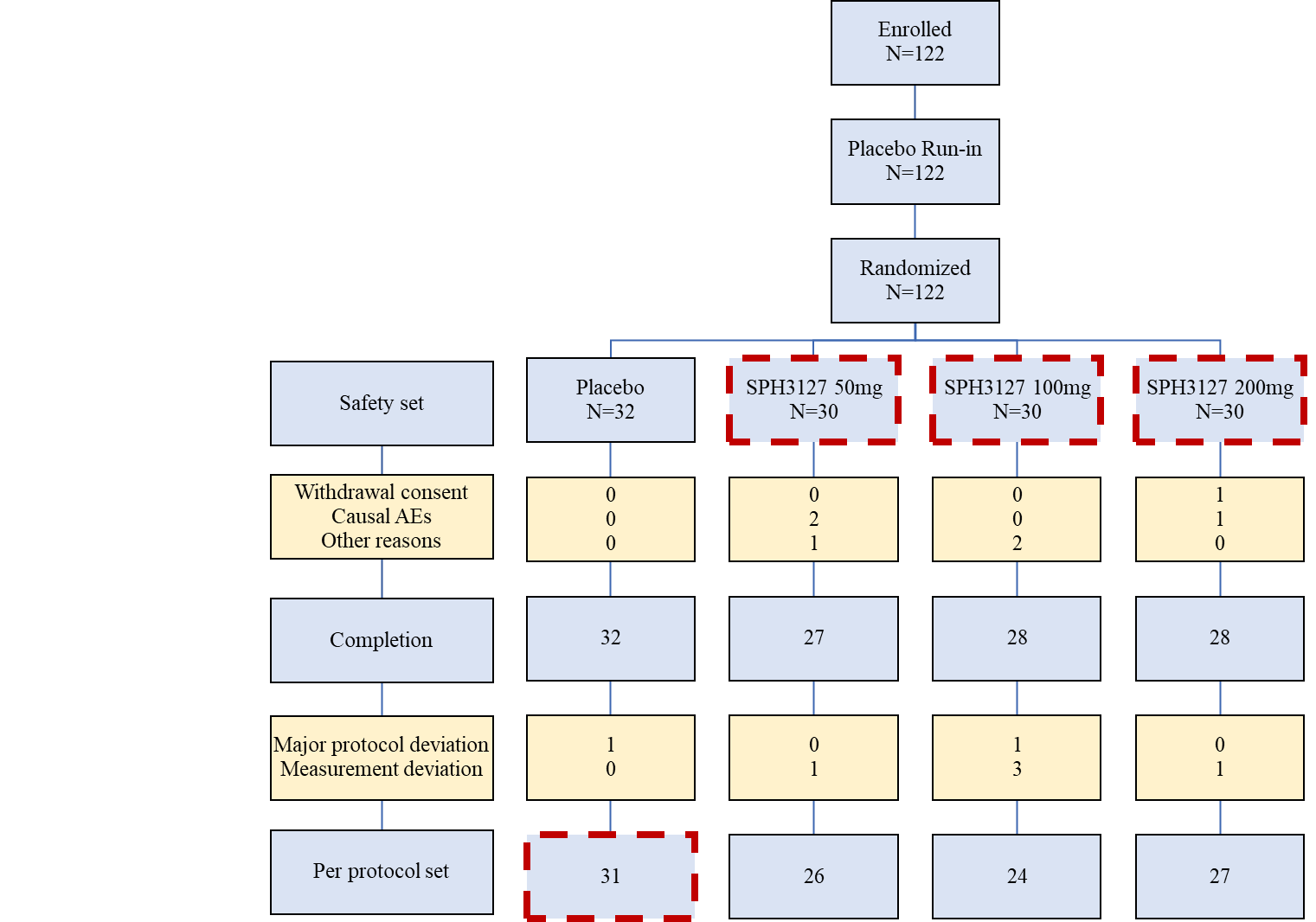
**

0

1

1

0

Major protocol deviation leading to exclusion from Per protocol set.

AE, adverse events

Annotation: Red boxes with intermittent border indicate the components of full analysis set (FAS).

**Supplementary Fig. 2** Results of ambulatory blood pressure monitoring. **(a)** 24-hour ambulatory msSBP; (b) 24-hour ambulatory msDBP.


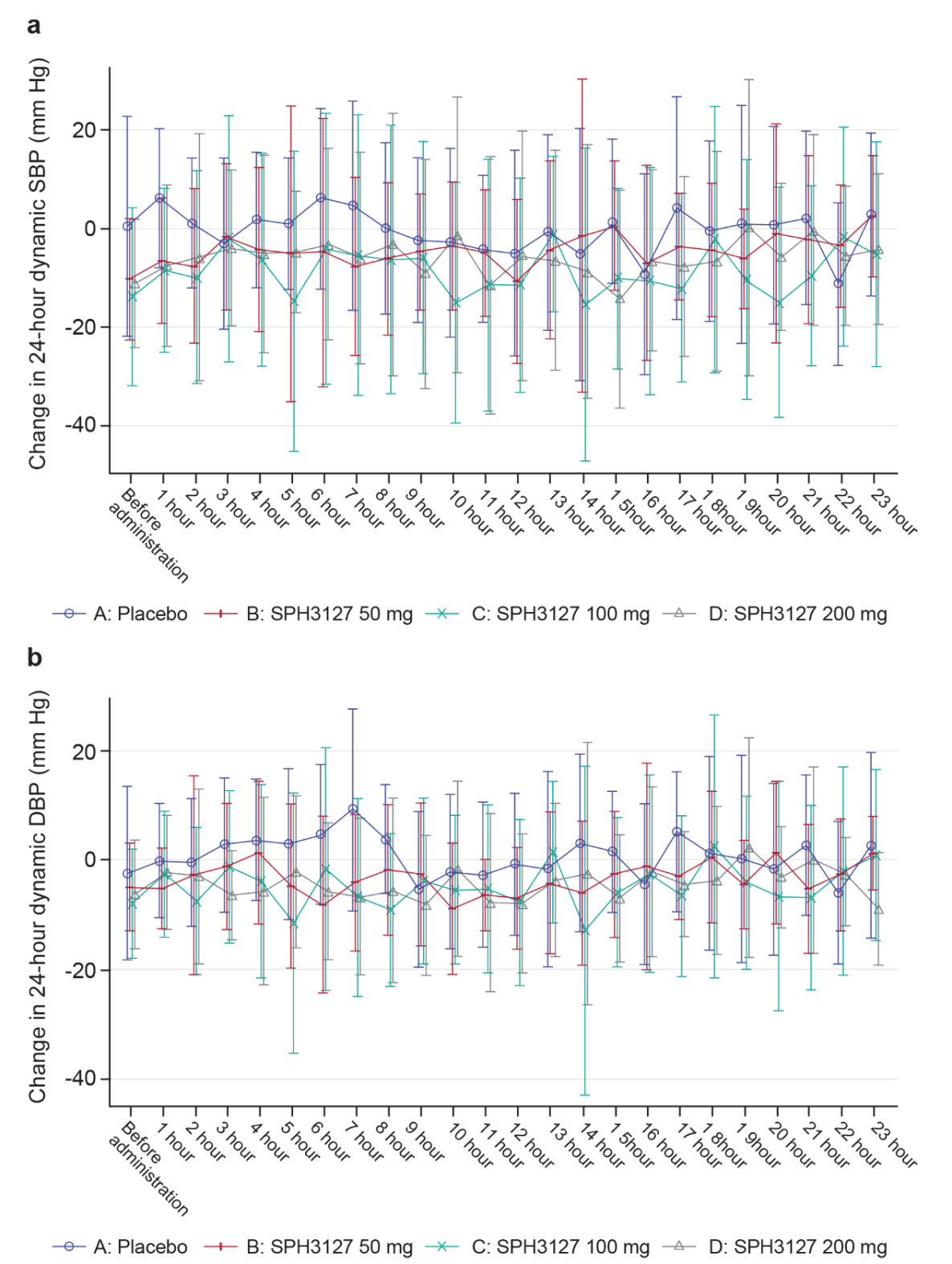


^DBP, diastolic blood pressure; SBP, systolic blood pressure^

**Supplementary Fig. 3** Change in plasma renin activity from baseline to 4 and 8 weeks of treatment


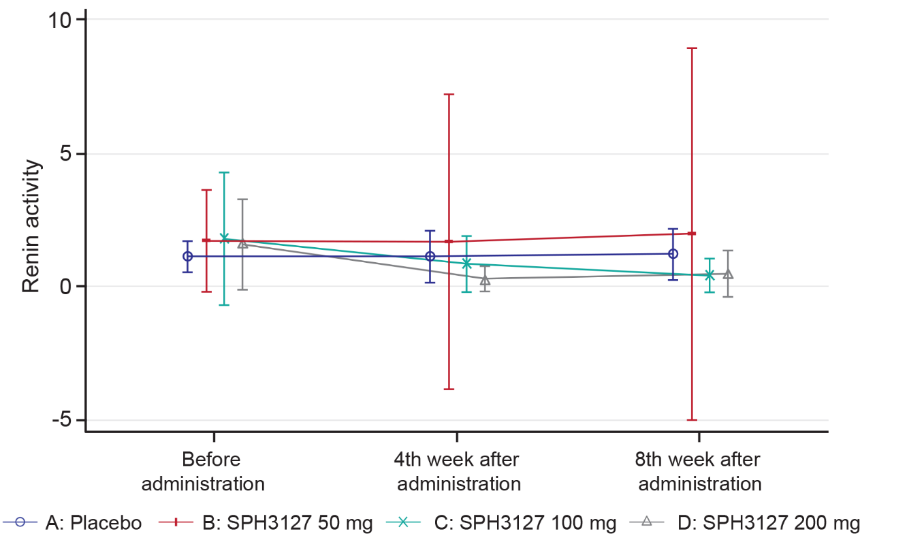


**Supplementary Table 1** The response rates at 4 and 8 weeks after treatment

|  | **FAS** | | | | **PPS** | | | |
| --- | --- | --- | --- | --- | --- | --- | --- | --- |
|  | **Placebo**  **(n = 31)** | **SPH3127 50 mg**  **(n = 30)** | **SPH3127**  **100 mg**  **(n = 30)** | **SPH3127**  **200 mg**  **(n = 30)** | **Placebo**  **(n = 31)** | **SPH3127**  **50 mg**  **(n = 26)** | **SPH3127**  **100 mg**  **(n = 24)** | **SPH3127**  **200 mg**  **(n =** **27)** |
| 4 weeks | | | | | | | | |
| Marked response, % | 12.90 | 23.33 | 30.00 | 23.33 | 12.90 | 26.92 | 33.33 | 22.22 |
| Response, % | 29.03 | 53.33 | 53.33 | 36.67 | 29.03 | 50.00 | 58.33 | 40.74 |
| Total response, % | 41.94 | 76.67 | 83.33 | 60.00 | 41.94 | 76.92 | 91.67 | 62.96 |
| Control rate, % | 25.81 | **53.33** | **56.67** | 40.00 | 25.81 | **53.85** | **66.67** | 40.74 |
| 8 weeks | | | | | | | | |
| Marked response, % | 22.58 | 23.33 | 33.33 | 30.00 | 22.58 | 26.92 | 41.67 | 29.63 |
| Response, % | 32.26 | 50.00 | 46.67 | 36.67 | 32.26 | 46.15 | 45.83 | 40.74 |
| Total response, % | 54.84 | 73.33 | **80.00** | 66.67 | 54.84 | 73.08 | **87.50** | 70.37 |
| Control rate, % | 41.94 | 50.00 | 63.33 | 60.00 | 41.94 | 50.00 | **75.00** | 62.96 |
| FAS, full analysis set, PPS, per protocol set  Total response rate = Marked response rate + Response rate.  Bold indicates the statistical difference in comparison with placebo. | | | | | | | | |
